# Self-care self-efficacy and depression associated with quality of life among patients undergoing hemodialysis in Vietnam

**DOI:** 10.1371/journal.pone.0270100

**Published:** 2022-06-16

**Authors:** Thi Thuy Nga Nguyen, Shu-Yuan Liang, Chieh-Yu Liu, Ching-Hui Chien

**Affiliations:** 1 Lecturer in Faculty of Nursing and Midwifery, Hanoi Medical University, Hanoi, Vietnam; 2 School of Nursing, National Taipei University of Nursing and Health Sciences, Taipei, Taiwan; 3 Department of Health Care Management, National Taipei University of Nursing and Health Sciences, Taipei, Taiwan; Faculty of Medicine, Saint-Joseph University, LEBANON

## Abstract

**Background:**

Hemodialysis impacts the quality of life of patients with end-stage renal disease. Particularly, depression is the most common psychological condition among patients. Self-care self-efficacy might play an important role in quality of life of patients with hemodialysis.

**Objective:**

This study was designed to explore the relationships among self-care self-efficacy, depression, and quality of life. The second aim was to explore the extent to which self-care self-efficacy and depression explain the variance in quality of life of patients on hemodialysis.

**Methods:**

This cross-sectional study included 127 patients receiving hemodialysis and used the Short Form 36 Health Survey, the Strategies Used by People to Promote Health, and the Patient Health Questionnaire 9 to evaluate quality of life, self-care self-efficacy, and depression. Data was analyzed using independent t-test, analysis of variance, Pearson’s correlation and hierarchical multiple regression.

**Results:**

The findings indicated that self-care self-efficacy was significantly positively correlated (PCS *r* = .533, *p *< 0.001, MCS *r* = .47, *p *< .001) and depression was significantly negatively correlated (PCS *r* = −.446, *p *< .001, MCS *r* = −.605, *p *< .001) with the two quality of life components. Self-care self-efficacy and depression were significant predictors of the physical (*R^2^inc* = 0.09, *β* = -0.38, *p*<0.001, *R^2^inc* = 0.12, *β =* -0.22, *p*<0.001) and mental (*R^2^inc* = 0.04%, *β* = -0.25, *p*<0.001, *R^2^inc* = 0.33, *β* = -0.51, *p*<0.001) quality of life of hemodialysis patients.

**Conclusions:**

Health professionals may target improving self-care self-efficacy and reducing depressive symptoms to enhance patient quality of life.

## Introduction

Chronic kidney disease (CKD) is becoming a global concern as global mortality related to CKD continues to grow. CKD is characterized by the progressive loss of kidney function, gradually leading to end-stage renal disease (ESRD), which requires kidney transplantation or dialysis [1]. In 2010, an estimated 2.0 million patients worldwide required hemodialysis [2], and Duong et al. (2015) estimated an annual increase of 7% [3]. The need for dialysis is projected to double by 2030, largely in developing regions of Asia and Africa [4]. In Vietnam, a developing economy, five million people suffer from kidney disease [5], and the prevalence of ESRD cases has continued to increase by approximately 8,000 new cases per year [5]. According to the director of Bach Mai Hospital’s Artificial Kidney Department, 1.3% of patients with ESRD eventually require hemodialysis [3].

Patients on hemodialysis must adhere to a strict treatment schedule as well as fluid and dietary restrictions [6, 7]. The psychological impact of such challenges may lead to depression [7], which is the most common psychological condition among patients with ESRD [8]. The prevalence of depression in the dialysis population ranges from 22.8% to 39.3% depending on whether interview-based diagnosis or self- or clinician-administered rating scales are used for assessment [8]. Depression correlates negatively with quality of life (QoL) [7–10]. Additionally, in a study by Perales-Montilla, worse depression predicted lower QoL, explaining approximately 50% of the variance in physical function, physical role, vitality, social function, and mental health on the Short Form 36 Health Survey (SF-36) scale [11].

Moreover, patients face a series of challenges in applying self-care strategies for the lifestyle modifications recommended with hemodialysis, such as diet and exercise [12]. Because of the difficulties that patients encounter with self-care at home, patient beliefs regarding their self-care ability play a key role in how they face treatment regimens. Patients who are confident in their ability to perform self-care behaviors are more likely to perform these behaviors [13]. That is, patients with higher self-efficacy are better able to adhere to prescribed medications or recommended diet and exercise plans suggested by health professionals [14]. This core belief in self-efficacy is the basis of human motivation, performance accomplishments, and emotional well-being [15]. Hence, increased self-efficacy is associated with increased treatment compliance, behaviors perceived as promoting health, and physical and psychological well-being [16].

Various studies have evaluated correlations between self-care self-efficacy and QoL or between psychological factors such as depression and QoL among patients receiving hemodialysis. In one study, lower levels of self-care self-efficacy were associated with the worst health-related QoL [1]. Moreover, research indicated that patients on hemodialysis who had high scores for depression were more likely to score low in QoL [7]. However, little research has examined the influence of both depression and self-care self-efficacy on the QoL of hemodialysis patients. In one study, self-care self-efficacy and depression were significant predictors of QoL after the effect of age was controlled [16]. That study was conducted almost 20 years ago, and the geographic area of the sample was limited to northern Taiwan. In Vietnam, a developing country, to the best of our knowledge, no prior studies have focused on the influence of self-care self-efficacy on the health-related QoL of patients on hemodialysis. Therefore, the purpose of this study was to explore the relationships among self-care self-efficacy, depression, and QoL. Another aim was to explore the amount of variance in the QoL among patients undergoing hemodialysis that could be accounted for by self-care self-efficacy and depression.

## Methods

### Sample and procedure

A cross-sectional survey was conducted with a convenience sample of adult Vietnamese patients (≥18 years old) undergoing hemodialysis for at least 3 months (it was considered a chronic situation that patients were necessary to adapt to the hemodialysis) at the Artificial Kidney Department at Bach Mai Hospital in Vietnam. Patients were able to read and write Vietnamese and agreed to participate in the study. Patients who had cognitive impairment, weakness and caregiver dependent were excluded from this study. The sample size was calculated by G-Power software based on a power of 0.80, an alpha of 0.05, and an effect size of 0.15 for linear multiple regression. Total sample size for the current study was 127.

Institutional review board (IRB) approval for the study was obtained from the IRB of Hanoi University of Public Health, Vietnam (no. 020-400/2020/DD-YTCC). The investigator conducted this study from October 2020 to June 2021. This research involved convenience sampling to include the patients during dialysis. The investigator explained the purpose and method of this study. After patients agreed to take part in this study and signed the consent form, the investigator invited patients to self-administering or interviewed patients to complete the questionnaires. It took approximately 20–30 minutes for patients to complete the questionnaires.

### Inclusivity in global research

Additional information regarding the ethical, cultural, and scientific considerations specific to inclusivity in global research is included in the S1 File.

### Measures

#### Sociodemographic variables

Patient self‐reported demographic data (age, gender, marital status, education level, monthly income, employment status) and medical characteristics (insurance status, dialysis vintage, and comorbidities) were collected.

#### Short Form 36 Health Survey

The Short Form 36 Health Survey (SF-36) is the most widely used generic instrument to estimate the QoL of patients on renal replacement therapy [17]. The SF-36 has been extensively used in CKD populations [18]. The questionnaire includes 1 item representing self-perceived changes in health and 35 items representing eight health domain scales yielding two summary measures: physical and mental health. The physical health measure (physical component summary, PCS) consists of four scales for physical functioning, role-physical, bodily pain, and general health. The mental health measure (mental component summary, MCS) includes vitality, social functioning, role-emotional, and mental health. A score from 0 to 100 is derived for each of PCS and MCS, with a higher score indicating better health status. The SF‐36 is available in Vietnamese, and the reliability of the Vietnamese version, with Cronbach’s α coefficients for the subscales ranging from .63 to .75 [19], is acceptable. Although Kidney Disease Quality of Life Instrument gives us more disease-specific aspects of QoL, the Vietnamese translation has not been evaluated and reviewed by RAND [20]. Therefore, we choose the SF-36 questionnaire to measure QoL among patients undergoing hemodialysis in this study.

#### Strategies used by people to promote health

Self-care self-efficacy is defined as psychological concept derived from self-efficacy that focuses on person’s confidence in his or her ability to perform relevant self-care activities [21]. Lev and Owen [21] developed the Strategies Used by People to Promote Health (SUPPH) to measure the self-care self-efficacy of patients with ESRD and cancer. This scale is reliable, with a Cronbach’s α for internal consistency of .93. Evidence of the convergent validity of the SUPPH was reported in terms of the correlation between the SUPPH and QoL (*r* = .34, *p *< .01) [21]. The SUPPH includes three subscales (stress reduction, decision making, and positive attitudes), with patients responding to 29 items. Items are rated on a 5-point scale of confidence from 1 (very little confidence) to 5 (quite a lot of confidence). The score is computed by calculating the mean of the responses to all items within each scale. Higher scores reflect better self-care self-efficacy. This study used a translation and back-translation process recommended by the WHO to produce a Vietnamese version of the SUPPH. All items have an item-level content validity index (I-CVI) of 1.00 and an average scale-level content validity index (S-CVI/Ave) of 1.00, which indicated that this questionnaire has good content validity. In this study, the reliability of the SUPPH (total scale) was high, with a Cronbach’s α of .95. The Cronbach’s α values for the three subscales, namely stress reduction, decision making, and positive attitudes, are .92, .71, and .92 respectively.

#### Patient Health Questionnaire 9

The Patient Health Questionnaire 9 (PHQ-9), developed by Dr. Robert Leopold Spitzer and colleagues in 2001 [22], includes nine symptom items. The questionnaire asks patients to report their symptoms over the last 2 weeks. Scores for items range from 0 (not at all) to 3 (nearly every day). A total score of 0 to 27 is calculated by summing the scores of the nine items, with a higher score representing more severe depression. A total score of 1 to 4 suggests minimal depression, 5 to 9 suggests mild depression, 10 to 14 suggests moderate depression, 15 to 19 suggests moderately severe depression, and 20 to 27 suggests severe depression. In this study, the reliability of the PHQ-9 was high, with a Cronbach’s alpha of .83.

### Statistical analysis

Data was analyzed using Statistical Package for the Social Sciences (SPSS, Chicago, IL, USA) 22. Descriptive statistics (mean (M), standard deviation (SD), frequency (n), and percentages (%)) are used to illustrate demographic and medical variables and levels of QoL, self-care self-efficacy, and depression. The associations among demographic/medical variables, self-care self-efficacy, depression, and QoL were assessed using Pearson’s correlations. Group differences for QoL were analyzed using independent-sample *t* tests and analysis of variance (ANOVA). Hierarchical multiple regression was used to determine how much variance (*R^2^inc*) in QoL could be accounted for by depression and self-care self-efficacy.

## Results

### Demographic characteristics

A total of 127 participants were recruited while undergoing hemodialysis in the Artificial Kidney Department of Bach Mai Hospital. Their age ranged from 21 to 84 years, with a mean age of 51.4 years. Most (52%) were female, married (76.4%), and had less than a high school education (69.3%). Only 40.9% were employed. Almost half had a monthly income of less than 5 million Vietnamese Dongs (VND) (n = 61, 48%; see Table 1).

**Table 1:** Demographic and medical characteristics (*n* = 127).

		n	%	Mean	SD
**Age** (years)	Range = 21–84			51.4	14.5
**Dialysis vintage** (months)				97.8	64.0
**Gender**	Male	61	48.0		
	Female	66	52.0		
**Employment status**	Unemployed	75	59.1		
	Employed	52	40.9		
** Marital status**	Unmarried	30	23.6		
	Married	97	76.4		
**Education level**	Less than high school	88	69.3		
	High school	27	21.3		
	University/College	12	9.4		
**Monthly income**	None income	48	37.8		
	< = 5 million dong	61	48.0		
	> 5 million dong	18	14.2		
**Insurance status**	Fully insured	83	65.4		
	Partially insured	44	34.6		
**Number of co-morbidities**	None	22	17.3		
	1	52	40.9		
	> = 2	53	41.7		

### Medical characteristics

The percentage of participants with full insurance coverage was 65.4%. Their average dialysis vintage was 97.8 months. Those with 1 comorbidity comprised 40.9% and ≥2 comorbidities comprised 41.7% of the participants (see Table 1).

### Status of QoL, self-efficacy, and depression

The scores on the MCS (M = 62.35, SD = 17.79) were higher than those for the PCS (M = 50.06, SD = 14.58). The self-care self-efficacy scale (the SUPPH total scale) indicated a moderate score of confidence in being able to perform self-care behaviors related to the illness (M = 2.96, SD = 0.66). The mean score on depression was 4.79 (SD = 4.47). Nearly half of the participants had minimal depression (n = 56, 44.1%), and just 0.8% had severe depression (see Table 2).

**Table 2:** Descriptive statistics for QoL, self-care self-efficacy (SUPPH scale), and depression (PHQ-9) (*n* = 127).

		n	%	Mean	SD
**Physical component summary (PCS)**				**50.06**	**14.58**
	Physical functioning			65.98	17.23
	Role functioning/physical			16.14	33.26
	Pain			76.91	25.97
	General health			41.22	13.73
**Mental component summary (MCS)**				**62.35**	**17.79**
	Role functioning/emotional			59.84	48.49
	Energy/fatigue			41.65	15.70
	Emotional well-being			66.90	19.65
	Social functioning			81.00	20.70
**SUPPH total scale**				**2.96**	**.66**
	Positive attitude			3.03	.65
	Stress reduction			2.86	.80
	Making decisions			2.92	.88
**PHQ-9**				**4.79**	**4.47**
**Depression level**	None	17	13.4		
	Minimal	56	44.1		
	Mild	38	29.9		
	Moderate	11	8.7		
	Moderately severe	4	3.1		
	Severe	1	.8		

### Associations between demographic and medical characteristics and QoL

Independent-sample *t* tests or ANOVA was used for differences between groups of variables. The results indicated a significant difference in physical QoL for patients according to employment status (*t* = 3.05, *p* = .003), insurance status (*t* = −1.99, *p* = .048), and monthly income (*F* = 6.42, *p* = .002). Moreover, a significant difference in mental QoL was observed according to employment status (*t* = −2.48, *p* = .015) and monthly income (*t* = 3.5, *p* = .033). Furthermore, the Pearson’s correlation results revealed that physical QoL had a significantly negative correlation with age (*r* = −.295, *p *< .001) and dialysis vintage (*r* = −.248, *p *< .001; see Table 3).

**Table 3:** Demographics and medical variables by PCS and MCS (n = 127).

			Physical component summary				Mental component summary			
Variables		n	Mean	SD	*t/F*	*p*-value	Mean	SD	*t/F*	*p*-value
**Gender**					-1.13	0.262			-1.78	0.078
	Female	66	48.7	14.7			59.7	17.9		
	Male	61	51.6	14.4			65.2	17.3		
**Employment status**					3.05	**0.003** **			-2.48	**0.015** *
	Employed	52	54.7	13.3			57.7	16.5		
	Unemployed	75	46.9	14.6			65.5	18.0		
**Marital status**					0.06	0.950			1.25	0.213
	Married	97	50.1	14.5			63.4	17.9		
	Unmarried	30	49.9	15.2			58.8	17.2		
**Insurance status**										
	Fully insured	83	48.2	13.8	-1.99	**0.048** *	61.1	17.7	-1.08	0.283
	Partially insured	44	53.6	15.5			64.7	17.9		
**Education level**					2.98	0.054			2.37	0.098
	Less than high school	88	48.2	13.6			60.4	17.7		
	High school	27	55.8	17.4			68.8	18.7		
	University/College	12	51.0	11.9			61.9	13.8		
**Monthly income**					6.42	**0.002** **			3.50	**0.033** *
	①None income	48	45.0	14.7		②,③>①	57.2	19.4		②>①
	②< = 5 million dong	61	51.8	14.3			66.1	16.1		
	③> 5 million dong	18	57.9	10.2			63.3	16.1		
**Number of co-morbidities**					2.03	0.136			0.12	0.885
	None	22	53.0	13.8			60.6	20.1		
	1	52	51.9	14.5			62.8	17.0		
	> = 2	53	47.0	14.6			62.6	17.9		

*p < .05

**p < .01

### Associations among self-care self-efficacy, depression, and QoL

Depression was significantly negatively moderately (PCS *r* = −.446, *p *< .001) to strongly (MCS *r* = −.605, *p *< .001) correlated with the two QoL components. Self-care self-efficacy had a significantly positively strong (PCS *r* = .533, *p *< 0.001) and moderate (MCS *r* = .47, *p *< .001) correlation with the components (see Table 4).

**Table 4:** Pearson correlations among demographics, depression (PHQ-9), self-care self-efficacy subscales (SUPPH subscales) and two components of QoL (PCS and MCS) (n = 127).

	PCS	MCS	Age	Duration	PHQ-9	SUPPH	Positive attitude	Stress reduction	Making decisions
**PCS**	1								
**MCS**	.416**	1							
**Age**	-.295**	.166	1						
**Dialysis vintage**	-.248**	.020	.154	1					
**PHQ-9**	-.446**	-.605**	.043	.111	1				
**SUPPH**	.533**	.470**	-.056	-.086	-.508**	1			
**Positive attitude**	.486**	.437**	-.076	-.077	-.465**	.972**	1		
**Stress reduction**	.513**	.468**	-.019	-.066	-.489**	.939**	.854**	1	
**Making decisions**	.434**	.309**	-.053	-.125	-.412**	.662**	.599**	.491**	1

**p < .001

### Self-care self-efficacy and depression predicting QoL

Hierarchical multiple regression explored how much of the variance in QoL among patients on hemodialysis could be accounted for by demographic and medical variables, self-care self-efficacy, and depression. In step one, significant demographic and medical variables were entered as predictors; in step two, depression was added as a predictor; in step three, self-care self-efficacy was added as a predictor. Self-care self-efficacy explained 9% of the variance (*R^2^inc* = .09, *p *< .001) in physical QoL, and depression explained 12% (*R^2^inc* = .12, *p *< .001; see Table 5). In mental QoL, self-care self-efficacy explained 4% of the variance (*R^2^inc* = .04, *p *< .001), and depression explained 33% *(R^2^inc* = .33, *p *< .001; see Table 6). The equation for predicting physical QoL (PCS) is intercept– 0.73⊆depression + 8.25⊆self-care self-efficacy. For every unit increased in depression, PCS is expected to decrease 0.73. For every unit increased in self-care self-efficacy, PCS is expected to increase 8.25. The equation for predicting mental QoL (MCS) is intercept– 2.04⊆depression + 6.75⊆self-care self-efficacy. For every unit increased in depression, MCS is expected to decrease 2.04. For every unit increased in self-care self-efficacy, MCS is expected to increase 6.75.

**Table 5:** Hierarchical multiple regression analysis for demographics, depression, and self-care self-efficacy (SUPPH) predicting PCS (*n* = 127).

PCS		Step 1			Step 2			Step 3		
Variables		B	SEB	β	B	SEB	β	B	SEB	β
**Age**		-.24	.09	**-.24** **	-.24	.08	**-.23** **	-.22	.08	**-.22** **
**Employment status**	Unemployed (ref)									
	Employed	2.68	2.81	.09	2.77	2.60	.09	2.70	2.42	.09
**Monthly income**	None income (ref)									
	< = 5 million d	5.51	2.70	**.19** *	2.05	2.60	.07	-.68	2.50	-.02
	>5 million d	7.26	4.17	.17	1.87	4.02	.05	-1.40	3.81	-.03
**Insurance status**	Partially insured (ref)									
	Fully insured	-3.82	2.57	-.13	-4.04	2.37	-.13	-2.66	2.23	-.09
**Dialysis vintage**		-.04	.02	-.16	-.03	.02	-.14	-.03	.02	-.14
**Depression**					-1.23	.26	**-.38** **	-.73	.27	**-.22** **
**SUPPH**								8.25	1.88	**.38** ***
**R^2^**inc			0.21			0.12			0.09	
**F**			5.5***			8.6***			11.1***	
**Overall model**	*R^2^* = 0.43 (*F*(8,118) = 11.09, *p*<0.001)									

*p < .05

**p < .01

***p < .001

**Table 6:** Hierarchical regression of SUPPH scale, depression scale, and demographics predicting MCS (*n* = 127).

MCS		Step 1			Step 2			Step 3		
Variables		B	SEB	β	B	SEB	β	B	SEB	β
**Employment status**	Unemployed (ref)									
	Employed	-12.51	3.32	**-.35** **	-12.37	2.62	**-.34** **	-12.47	2.52	**-.35** **
**Monthly income**	None income (ref)									
	< = 5 million d	12.26	3.33	**.35** **	5.32	2.74	.15	3.01	2.73	.09
	>5 million d	14.10	5.06	**.28** *	3.15	4.18	.06	.12	4.13	.00
**Depression**					-2.45	.28	**-.61** **	-2.04	.30	**-.51** **
**SUPPH**								6.70	2.06	**.25** **
**R^2^**inc			0.15			0.33			0.04	
**F**			7.3**			27.9**			26.2**	
**Overall model**	*R^2^* = 0.52 (*F*(5,121) = 26.20, *p*<0.001)									

*p < .01

**p < .001

## Discussion

In this study, the QoL of patients undergoing hemodialysis was slightly low. This finding is in line with studies [10, 23, 24] that have concluded that QoL among patients undergoing hemodialysis was lower than in the general population. People on hemodialysis have lower QoL for several reasons. Hemodialysis extends the lives of patients with ESRD; however, they frequently experience complications and dietary limitations [6, 7]. Moreover, they face psychological challenges, such as anxiety, worry, sadness, depression, loss of control, and fear of death [7].

Patients in this study demonstrated a moderate level of self-efficacy regarding their ability to perform self-care behaviors related to their illness. This self-efficacy level is similar to the results of studies conducted in Iran, where the same instrument has been widely used [23, 25, 26]. Moreover, in the present study, scores were lower in the stress reduction and decision-making dimensions but higher in the dimension of positive attitude. The low score for decision-making self-efficacy may be due to the fact patients are often strongly dependent on decisions and actions of health care professionals [27]. In Vietnam, although doctors do offer treatment options, many patients prefer to have doctors or family members make decisions regarding treatment. Most Vietnamese adults are married and live in multiple-generation families, thus, they often receive strong social support, and families are commonly involved in medical decision making [28]. Furthermore, education improves patient self-care self-efficacy [29]. However, in the current study, most patients had a low education level, which might (partially) explain the relatively low level of self-care self-efficacy.

Patients undergoing hemodialysis experienced low to severe levels of depression, which were higher than prevalence estimates reported in other studies [10, 30–32]. In Vietnamese culture, most people live with family members and support each other. When they become ill, their self-care ability declines, and they may feel that they are an economic and caregiving burden on the family [28]. Moreover, the current study was conducted during the COVID-19 pandemic, which caused numerous forms of stress and may be a cause of the relatively stronger depressive symptoms [33].

Self-care self-efficacy had a significantly positive correlation with all components of the SF-36 QoL scale, meaning that those with higher self-care self-efficacy tended to have higher QoL. In addition, the three subscales of the SUPPH were significantly positively correlated with all the components of the SF-36 scale. This result supports the findings of previous studies in which patients who had higher self-care self-efficacy had higher QoL [1, 6, 23, 34, 35]. Self-efficacy is associated with self-care for patients on hemodialysis. When individuals perceive self-efficacy, they believe in their capacity to take care of themselves. According to Bandura (1986), self-efficacy mediates perceptions of health-related QoL [36]. The evidence demonstrates a need for enhancing the self-care self-efficacy of patients on hemodialysis to improve their QoL.

Depression had a significant negative correlation with all components of the SF-36 QoL scale. This result supports reported findings indicating that patients who have more severe depression have lower QoL [7, 24, 32]. The reason might be that patients on hemodialysis often experience uncomfortable symptoms such as itching, sexual dysfunction, and physical limitations. They may also feel they are an economic burden due to job loss and low incomes. Caregivers reported a high level of burden in caring individuals undergoing hemodialysis [28]. Moreover, vascular access and blood collection for laboratory examination three times a week can be painful [37]. All these elements can burden patients and contribute to depression. Identifying and measuring depressive symptoms is vital because they may contribute to a lower QoL.

When we investigated details of physical and mental health, the results indicated that self-care self-efficacy is a prominent predictor of QoL. Depression was a stronger predictor (33% of variance) of the mental aspects of QoL than self-care self-efficacy was (4% of variance), which is unsurprising because depression is a dimension of mental health. However, self-care self-efficacy still had a key role; adding that variable to the model increased the predictive variance from 48% to 52%. In physical QoL, the difference in predictive variance of depression (12% of variance) and self-care self-efficacy (9% of variance) was not as great. Moreover, the standardized coefficient for self-care self-efficacy (*β* = .38, *p *< .001) was higher than that for depression (*β* = −.22, *p *< .001). Therefore, our result refines the conclusions of researchers who have reported that self-care self-efficacy is a key predictor of QoL among patients on hemodialysis [1, 35]; the present study suggests that it is related to physical aspects of QoL in particular. Depression was a key variable predicting patient QoL. A study by Ganu et al. (2018) likewise determined that depression was a significant factor predicting overall QoL [7]. More specifically, in a study by Perales-Montilla et al. (2012), worse depression predicted lower QoL, explaining approximately 50% of the variance in physical function, physical role, vitality, social function, and mental health on the SF-36 scale [11]. Our result extends the work of previous researchers reporting that depression is a prominent predictor of the QoL of hemodialysis patients.

In contrast with the results of this study, a study conducted in Taiwan concluded that self-care self-efficacy was a far more powerful predictor than depression was, with self-care self-efficacy predicting 47.5% of the variance and depression explaining only 5.5% of the variance in overall QoL after the effect of age was controlled [16]. The Taiwan study used the same instrument for self-efficacy but different instruments for QoL and depression. Their QoL questionnaire combined physical and mental health, whereas we separated the physical component of QoL from the mental component. We explored the degree to which self-care self-efficacy could predict aspects of physical and mental health. Moreover, in its regression, the Taiwan study used self-care self-efficacy as a variable in step two and included depression in step three, whereas we used self-care self-efficacy in step three because self-efficacy was the variable of greatest interest. Many statistics experts advise researchers to add the variable they want to explore in the final step of the regression [38, 39]. Including the variable of greatest interest in the final step of the regression puts the focus on the change in predictability associated with predictor variables entered later in the analysis over and beyond that contributed by predictor variables entered earlier in the analysis.

Self-care self-efficacy is useful for predicting QoL. The finding advances nursing research and is supported by previous work. Moreover, this study provides evidence from a developing country in Southeast Asia. Clinically, the study points to a need to evaluate the self-care self-efficacy of hemodialysis patients experiencing poor QoL. Health professionals should support the self-care strategies of individuals with hemodialysis and design interventions to enhance patient confidence in their self-care ability. Particularly, e health serves might be one of the important strategies for telemedicine and care during and after pandemic. Self-care self-efficacy therefore may play a crucial role in QoL of patients at home care.

This cross-sessional study used convenience sampling from a single dialysis center in northern Vietnam. The focus was limited to outpatients receiving hemodialysis, which probably affects the generalizability of the study. This study cannot support the causalities among the variables of self-care self-efficacy, depression, and QoL.

## Conclusions

The findings revealed that self-care self-efficacy and depression were significant predictors of QoL among patients on hemodialysis. Patients undergoing hemodialysis in Bach Mai Hospital in Vietnam do not have adequate self-care self-efficacy and report low QoL. Health professionals should design interventions to enhance patient confidence in their self-care ability to improve their QoL.

## Supporting information

S1 File (DOCX)

S1 Data (SAV)
